# Association between red cell distribution width to albumin ratio and clinical outcomes in elderly patients with sepsis: a cohort study

**DOI:** 10.3389/fnut.2025.1617199

**Published:** 2025-09-02

**Authors:** Li An, Zhiqing Fu, Zhenhong Chen, Xiaomiao Xiong, Minsheng Li, Limei Lu, Zhijian Zhang, Shan Li

**Affiliations:** ^1^Department of Respiratory and Critical Care Medicine, The Second Medical Center and National Clinical Research Center for Geriatric Disease, Chinese PLA General Hospital, Beijing, China; ^2^Department of Cardiology, The Second Medical Center and National Clinical Research Center for Geriatric Disease, Chinese PLA General Hospital, Beijing, China; ^3^Department of Respiratory and Critical Care Medicine, Hainan Hospital of Chinese PLA General Hospital, Sanya, China; ^4^The Third Department of Healthcare, The Second Medical Center and National Clinical Research Center for Geriatric Disease, Chinese PLA General Hospital, Beijing, China

**Keywords:** sepsis, elderly, red cell distribution width, albumin, mortality

## Abstract

**Background:**

This study investigates the association between red cell distribution width to albumin ratio (RAR) and clinical outcomes in elderly sepsis patients.

**Methods:**

This study investigates the association between red cell distribution width to albumin ratio (RAR) and clinical outcomes in elderly sepsis patients. Methods: Using the eICU-CRD (2014–2015), 5,976 sepsis patients aged≥60 years were stratified into RAR quartiles at ICU admission: Q1 (≤5.28), Q2 (5.29–6.37), Q3 (6.38–7.87), and Q4 (7.88–15.0), with Q1 as the reference category. The primary outcome was 28-day hospital mortality, while secondary outcomes included ICU mortality, 90-day hospital mortality, and lengths of ICU and hospital stays. Multivariable regression analysis and spline curves from the generalized additive model were applied to assess the association between RAR and clinical outcomes. Kaplan–Meier survival analysis illustrated cumulative hospital mortality across RAR quartiles.

**Results:**

The 28-day hospital, ICU, and 90-day hospital mortality were 17.4, 10.9, 17.8%, respectively, with ICU and hospital stays of 2.9 (1.8–5.1) and 7.1 (4.6–11.7) days. Compared to Q1, Q4 exhibited significantly increased risks of 28-day hospital mortality (adjusted odds ratio [OR]: 2.95, 95% confidence interval [CI]: 2.28–3.80), ICU mortality (adjusted OR: 2.06, 95% CI: 1.52–2.78), 90-day hospital mortality (adjusted OR: 3.03, 95% CI: 2.35–3.90), and prolonged ICU (*β*: 0.89, 95% CI: 0.42, 1.36) and hospital stays (β: 1.64, 95% CI: 0.93, 2.36). Generalized additive model revealed linear relationship between RAR and mortality. Kaplan–Meier survival analysis demonstrated higher mortality with elevated RAR quartiles.

**Conclusion:**

Elevated baseline RAR is independently associated with adverse clinical outcomes in elderly sepsis patients, suggesting it may be a valuable tool for early risk stratification and personalized therapeutic interventions.

## 1 Introduction

Sepsis continues to be a critical global health burden, contributing substantially to both morbidity and mortality. According to the 2017 Global Burden of Disease (GBD) study, sepsis accounted for an approximately 48.9 million cases worldwide and resulted in 11.0 million sepsis-related deaths, constituting 19.7% of all global fatalities (1). Elderly populations exhibit a significantly increased susceptibility to sepsis and its associated complications, with a 13.1-fold elevation in the relative risk of sepsis-related mortality compared to younger individuals (2). This heightened vulnerability is due to age-related pathophysiological alterations such as immunosenescence, multimorbidity, chronic low-grade inflammatory state, sarcopenia and malnutrition (3–5). Despite advances in sepsis management, there remains a critical unmet need for early risk stratification and personalized therapeutic strategies to improve prognostic outcomes in elderly sepsis patients. However, timely assessment of elderly sepsis patients is often hindered by their atypical clinical presentations (6), highlighting the demand for robust biomarkers to facilitate early risk stratification and optimize therapeutic interventions.

The red cell distribution width (RDW), a routine component of complete blood count (CBC), quantifies erythrocyte size heterogeneity. Beyond its traditional role in anemia diagnosis, elevated RDW has emerged as a biomarker reflecting the composite effects of systemic inflammation (7), oxidative stress (8, 9), and sepsis-related hemolysis (10, 11). These pathological processes collectively lead to dysregulated erythropoiesis, erythrocyte membrane destabilization, and erythrocyte destruction, ultimately resulting in increased red blood cell size heterogeneity. Emerging evidence has further elucidated a significant association between RDW and heightened mortality across multiple pathological conditions, including cardiovascular diseases, malignancies, and sepsis (12–14). Plasma albumin, the most predominant circulating protein, maintains physiological homeostasis through osmotic regulation, antioxidant activity, and anti-inflammatory modulation (15). Hypoalbuminemia has been extensively linked to adverse clinical outcomes across various diseases (16–18). In the context of sepsis, hypoalbuminemia is associated with exacerbated inflammation, organ dysfunction, and mortality risk (19, 20).

The red cell distribution width to albumin ratio (RAR) represents a novel composite index derived by dividing RDW by albumin, a simple, cost-effective, and readily accessible parameter in clinical practice. RAR has gained considerable attention in recent research due to its unique ability to reflect both erythrocyte dysfunction and hypoalbuminemia. Elevated RAR has been associated with increased mortality across various critical illnesses, including sepsis (13, 21–24). However, existing studies predominantly focus on adult patients, with limited investigation into the relationship between RAR and prognosis in elderly patients with sepsis. Therefore, further research is warranted to clarify the specific association between RAR and sepsis outcomes in this vulnerable population.

To address this gap, we investigated the relationship between RAR levels and mortality risk, along with the duration of ICU and hospital stays, by analyzing data from elderly sepsis patients in the multicenter eICU database.

## 2 Materials and methods

### 2.1 Data source and study population

This study was a retrospective observational analysis utilizing data extracted from the publicly accessible eICU Collaborative Research Database (eICU-CRD, version 2.0). The eICU-CRD is a multicenter ICU database that encompasses comprehensive clinical data from over 200,000 admissions across 208 hospitals in the United States during the 2014–2015 period (25). Owning to its retrospective design, the absence of direct patient intervention, and compliance with a security schema certified by Privacert (Cambridge, MA) as meeting safe harbor standards for reidentification risk, the study was exempt from institutional review board approval at the Massachusetts Institute of Technology. Consequently, informed consent was also waived. LA obtained database access and extracted all data records (ID: 60127551).

**Figure 1:**
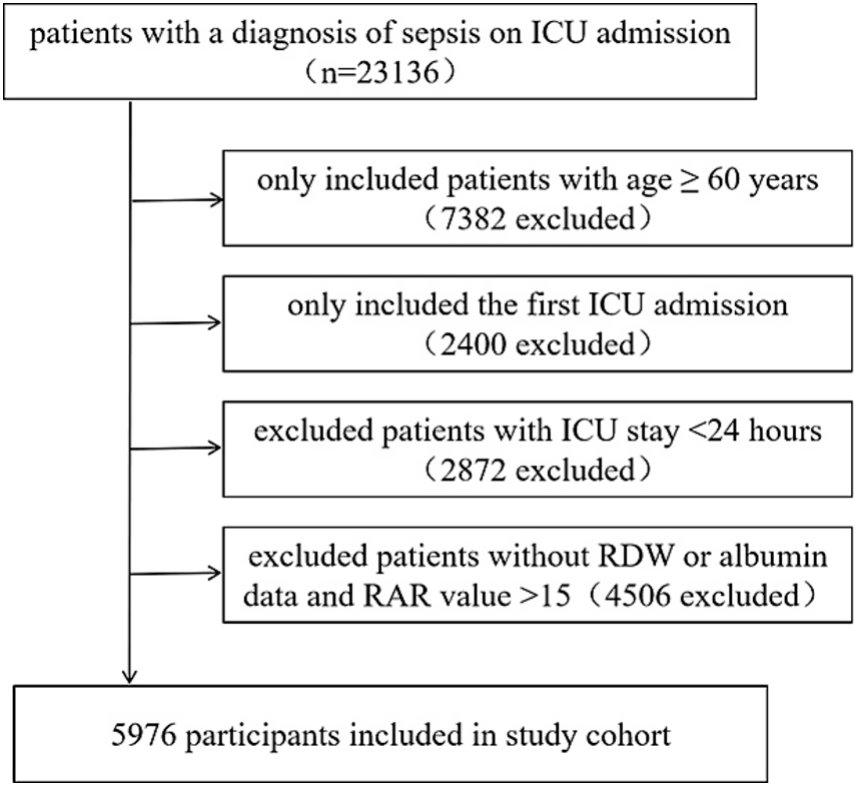
Flow chart of the study population.

All patients who fulfilled the criteria for sepsis upon admission to the Intensive Care Unit (ICU) were included in the study. Sepsis was defined as the presence of a suspected or confirmed infection, coupled with an acute increase in the Sequential Organ Failure Assessment (SOFA) score exceeding 2 points, as recorded in the Acute Physiology and Chronic Health Evaluation (APACHE) IV dataset. Infection identification was performed using the ICD-9 code within the eICU-CRD. The exclusion criteria were as follows: (1) patients with a SOFA score < 2; (2) age < 60 years; (3) not the first ICU admission; (4) ICU stay duration < 24 h; and (5) missing data on RDW or albumin following ICU admission; (6) extreme RAR values (>15) indicative of measurement outliers. The enrollment flowchart is presented in Figure 1.

### 2.2 Measurement of RAR

The baseline RAR (%/g/dL) was calculated as the ratio of RDW (%) to serum albumin (g/dL), using the first laboratory measurements obtained upon ICU admission. Patients were stratified into quartiles based on RAR: Q1 (≤5.28, *n* = 1,492), Q2 (5.29–6.37, *n* = 1,495), Q3 (6.38–7.87, *n* = 1,494), and Q4 (≥7.88, *n* = 1,495), with Q1 serving as the reference category.

### 2.3 Outcomes

The primary outcome was 28-day all-cause hospital mortality. Secondary outcomes included ICU mortality, 90-day all-cause hospital mortality, ICU length of stay, and hospital length of stay.

### 2.4 Covariates

Covariates were selected based on clinical experience and on a > 10% change in the exposure’s effect estimate upon adjustment. All measurements were obtained within the initial 24 h post-ICU admission. The final set included: (1) Demographics: gender, age, ethnicity; (2) Physiological variables: body mass index (BMI), SOFA score, and Simplified Acute Physiology Score (SAPS)II; (3) Comorbidities and interventions: congestive heart failure (CHF), acute myocardial infarction (AMI), hepatic failure, malignancy, diabetes mellitus (DM), chronic obstructive pulmonary disease (COPD), mechanical ventilation, dialysis, and vasopressor use; (4) Laboratory parameters: blood urea nitrogen (BUN), serum creatinine (Scr), bicarbonate, glucose, alanine transaminase (ALT), aspartate transaminase (AST), total bilirubin, hemoglobin (Hb), platelet count (PLT), and white blood cell count (WBC).

### 2.5 Statistical analysis

Continuous variables were presented as mean ± standard deviation (SD) for normally distributed data or median with interquartile ranges (IQR) for non-normally distributed data. Categorical data were summarized as frequencies and corresponding percentages. Comparisons among RAR quartile groups were conducted using one-way ANOVA for normally distributed continuous variables (normality verified by Shapiro–Wilk test and homogeneity of variance confirmed by Levene’s test) or Kruskal-Wallis test for non-normally distributed continuous variables. Group differences in categorical variables were evaluated using chi-square tests.

Multivariable logistic and linear regression models were used to evaluate the associations between RAR and clinical outcomes, presenting adjusted odds ratio (OR) with 95% confidence interval (CI) for mortality and βcoefficients with 95% CI for ICU/hospital length of stay. RAR was examined both as a continuous variable (reporting effects per 1-unit increment and per standard deviation increase to account for extreme values) and categorically by quartiles. Additionally, trend analyses were conducted across ordinal quartile levels. Three distinct models were constructed: ModelIwas unadjusted; ModelII was adjusted for age, gender, and ethnicity; model III was further adjusted for BMI, SAPSII, SOFA score, mechanical ventilation, dialysis, vasopressor use, hepatic failure, metastatic cancer, immunosuppression, DM, COPD, CHF, AMI, site of infection, BUN, creatinine, bicarbonate, glucose, ALT, AST, platelets, hemoglobin, WBC. Generalized additive model with spline curves were used to explore the association between RAR and the mortality outcomes. Kaplan–Meier analysis with log-rank tests was conducted to evaluate cumulative 28-day and 90-day hospital mortality across RAR quartiles.

Missing values for the covariates were as follows: BMI (126, 2.2%), SAPSII (705, 13.3%), BUN (11, 0.2%), creatinine (23, 0.4%), bicarbonate (663,12.5%), glucose (54, 0.9%), AST (450, 8.1%), ALT (507, 9.2%), platelet (33, 0.6%), hemoglobin (8, 0.1%), WBC (10, 0.2%). Dummy variables were used to represent missing covariate values.

All statistical analyses were performed using EmpowerStats (X&Y Solutions, Inc., Boston, Massachusetts) and R software (version 4.2.0), with statistical significance defined as *p* < 0.05 (two-sided). Subgroup analyses were conducted using multivariable logistic regression, with stratification based on gender (male or female), age (≤70, 70–80, >80), SOFA score (≤4, 5–8, ≥9), mechanical ventilation (yes or no), dialysis (yes or no), vasopressor use (yes or no), infection site (pulmonary, urinary, gastrointestinal, others), CHF (yes or no), and DM (yes or no). Furthermore, three sensitivity analyses were conducted to evaluate the robustness of the primary findings. First, we performed a sensitivity analysis restricted to complete cases to mitigate potential bias arising from missing data. Secondly, to assess the potential impact of transfusions involving red blood cells, plasma, or human serum albumin on RAR, a sensitivity analysis was performed. This analysis excluded patients who had received any of the aforementioned transfusions within the 2 days preceding their admission to the ICU. Thirdly, to assess the potential effect modification by anemia on RAR, the cohort was stratified into two groups: anemia and non-anemia, based on the World Health Organization’s definition of anemia (26). We then conducted multivariable regression analyses for each group separatedly.

## 3 Results

### 3.1 Baseline characteristics

The participants screening strategy is illustrated in Figure 1. Ultimately, a total of 5,976 elderly patients with sepsis were included in this study, with a mean age of 74.4 ± 9.0, of whom 2,908 (48.7%) were male. The study endpoints revealed that 28-day hospital, ICU, 90-day hospital mortality were 1,041 (17.4%), 653 (10.9%), 1,062 (17.8%), respectively. The length of stay in the ICU and hospital were 2.9 (1.8–5.1) days and 7.1 (4.6–11.7) days, respectively. The baseline characteristics of the patient cohort are presented in Table 1. RAR value ranged from 2.49 to 15.00, with a mean of 6.82 ± 2.10%/g/dL. There was an increasing trend in SOFA, SAPS II scores, BUN, creatinine, AST, and WBC, as well as in the prevalence of hepatic failure, immunosuppression, metastatic cancer, ventilation, vasopressor use, and dialysis from Q1 to Q4 of RAR. Conversely, there was a decreasing trend in BMI, bicarbonate, hemoglobin, as well as the prevalence of CHF and AMI.

**Table 1:** Baseline characteristics and outcomes of participants categorized by RAR.

Variables	RAR				*p* value
	Q1, *n* = 1,492 ≤ 5.28	Q2, *n* = 1,495 5.29–6.37	Q3, *n* = 1,494 6.38–7.87	Q4, *n* = 1,495 7.88–15.00	
Gender, *n* (%)					0.061
Male	696 (46.7)	769 (51.4)	716 (47.9)	727 (48.6)	
Female	796 (53.3)	726 (48.6)	778 (52.1)	768 (51.4)	
Age (years)	74.6 ± 9.0	75.2 ± 9.1	74.6 ± 9.0	73.4 ± 8.6	<0.001
Ethnicity, *n* (%)					0.002
White	1,241 (83.2)	1,198 (80.1)	1,220 (81.7)	1,165 (77.9)	
Others	251 (16.8)	297 (19.9)	274 (18.3)	330 (22.1)	
BMI (kg/m^2^)	29.1 ± 8.2	28.6 ± 8.0	27.9 ± 8.0	27.6 ± 7.9	<0.001
SAPS II score	52.8 ± 21.0	57.3 ± 22.9	62.0 ± 23.7	68.9 ± 25.0	<0.001
SOFA score	4.0 (2.0–6.0)	4.0 (2.0–7.0)	5.0 (3.0–7.0)	5.0 (3.0–8.0)	<0.001
Hepatic failure, *n* (%)	12 (0.8)	23 (1.6)	19 (1.3)	45 (3.0)	<0.001
Immunosuppression, *n* (%)	53 (3.6)	74 (5.0)	95 (6.4)	121 (8.2)	<0.001
Metastatic cancer, *n* (%)	20 (1.4)	34 (2.3)	61 (4.1)	98 (6.6)	<0.001
CHF, *n* (%)	177 (11.9)	145 (9.7)	146 (9.8)	126 (8.4)	0.018
AMI, *n* (%)	82 (5.5)	71 (4.8)	52 (3.5)	44 (2.9)	0.002
DM, *n* (%)	219 (14.7)	213 (14.3)	211 (14.1)	214 (14.3)	0.976
COPD, *n* (%)	153 (10.3)	143 (9.6)	135 (9.0)	111 (7.4)	0.048
infection site, *n* (%)					<0.001
Pulmonary	627 (42.0)	588 (39.3)	544 (36.4)	545 (36.5)	
Urinary	364 (24.4)	432 (28.9)	394 (26.4)	321 (21.5)	
Gastrointestinal	178 (11.9)	176 (11.8)	221 (14.8)	244 (16.3)	
Others	323 (21.6)	299 (20.0)	335 (22.4)	385 (25.8)	
Ventilator use, *n* (%)	404 (27.5)	425 (28.8)	430 (29.1)	486 (32.8)	0.012
Dialysis, *n* (%)	69 (4.7)	68 (4.6)	68 (4.6)	104 (7.0)	0.005
Vasopressor, *n* (%)	375 (25.2)	482 (32.2)	517 (34.6)	618 (41.5)	<0.001
BUN (mg/dL)	28 (19–44)	31 (20–47)	33 (22–52)	35 (22–57)	<0.001
Creatinine (mg/dL)	1.4 (0.9–2.3)	1.5 (1.0–2.4)	1.5 (1.0–2.5)	1.6 (1.0–2.7)	0.034
Bicarbonate (mmol/L)	22.9 ± 5.1	22.6 ± 5.5	22.2 ± 5.4	21.5 ± 5.4	<0.001
Glucose (mg/dL)	138 (111–184)	129 (103–172)	127 (100–166)	124 (99–168)	<0.001
AST (U/L)	32 (20–65)	34 (21–70)	34 (21–74)	39 (22–77)	0.002
ALT (U/L)	26 (16–47)	27 (16–51)	26 (16–54)	27 (16–51)	0.829
Total bilirubin (mg/dL)	0.7 (0.4–1.0)	0.7 (0.4–1.1)	0.7 (0.4–1.2)	0.7 (0.4–1.4)	<0.001
Platelets, (cells x 10^9^/L)	178 (130–242)	176 (124–236)	178 (121–255)	184 (112–277)	0.338
Hemoglobin, (g/dL)	11.5 ± 2.0	10.5 ± 1.9	10.1 ± 1.9	9.4 ± 1.9	<0.001
WBC, (cells x 10^9^/L)	13.0 (9.0–17.9)	13.1 (9.1–19.1)	13.8 (9.0–20.6)	14.6 (8.7–21.5)	<0.001
28-day hospital mortality, *n* (%)	135 (9.1)	183 (12.2)	284 (19.0)	439 (29.4)	<0.001
ICU mortality, *n* (%)	94 (6.3)	119 (8.0)	160 (10.7)	280 (18.7)	<0.001
90-day hospital mortality, *n* (%)	136 (9.1)	188 (12.6)	288 (19.3)	450 (30.1)	<0.001
LOS in ICU, (day)	2.7 (1.8–4.4)	2.8 (1.8–5.0)	2.9 (1.8–5.2)	3.3 (1.9–5.9)	<0.001
LOS in hospital, (day)	6.6 (4.3–10.0)	7.0 (4.6–11.4)	7.5 (4.9–12.0)	7.8 (4.5–13.1)	<0.001

### 3.2 Associations between RAR and clinical outcomes

**Figure 2:**
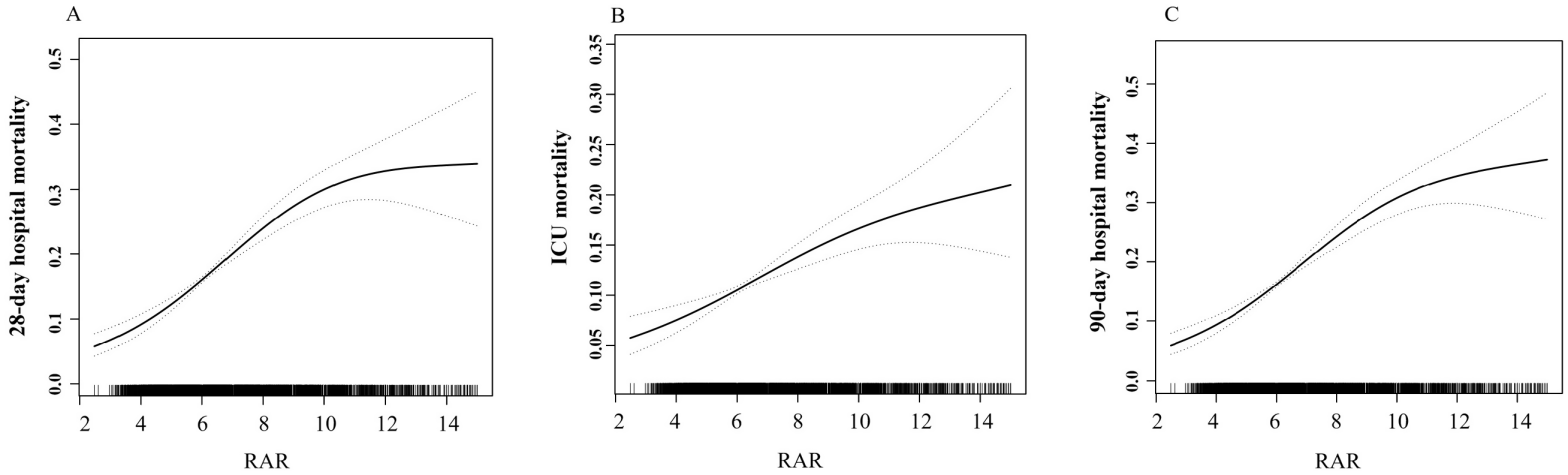
Association between RAR and risks of mortality. **(A)** 28-day hospital mortality. **(B)** ICU mortality. **(C)** 90-day hospital mortality.

**Figure 3:**
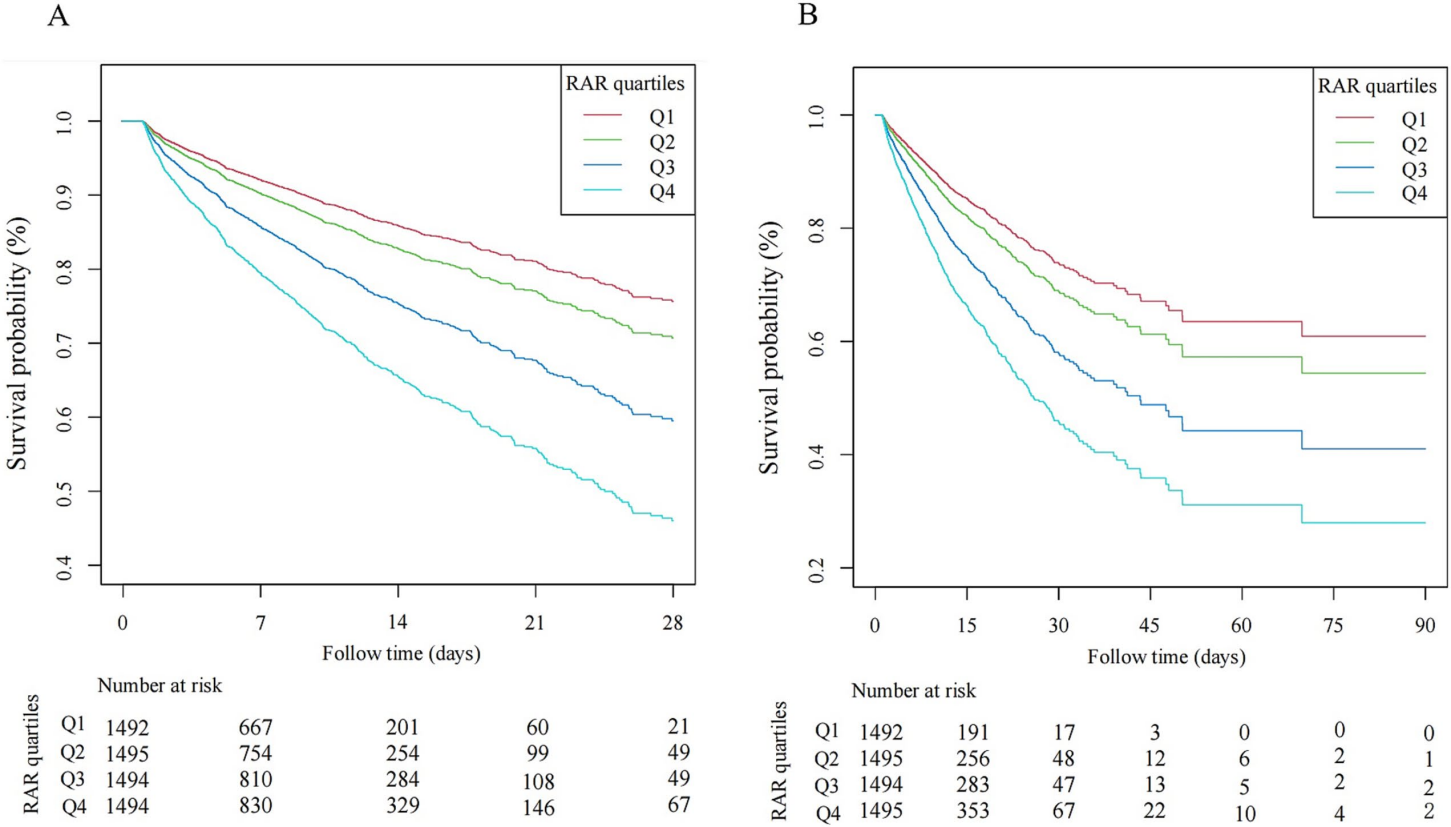
Kaplan–Meier curve of hospital mortality with sepsis. **(A)** 28-day hospital mortality. **(B)** 90-day hospital mortality.

Compared with Q1 group, patients in higher quartiles exhibited significantly increased mortality. Specifically, the adjusted OR for Q4 versus Q1 were 2.95 (95% CI 2.28–3.80) for 28-day hospital mortality, 2.06 (1.52–2.78) for ICU mortality, 3.03 (2.35–3.90) for 90-day hospital mortality. The *β* coefficients for Q4 versus Q1 were 0.89 (0.42–1.36) for ICU length of stay and 1.64 (0.93–2.36) for hospital length of stay. A dose-dependent increase in mortality risks and lengths of stay in the ICU and hospital was observed across ascending RAR quartiles, with all trend *p*-value< 0.05. Similar results were obtained when RAR was analyzed as a continuous variable, based on both unit and standard deviation (SD) increases (Table 2). Moreover, spline curves derived from generalized additive model demonstrated a linear increase in the risks of 28-day hospital mortality, ICU mortality, and 90-day hospital mortality with rising RAR levels (Figure 2). Kaplan–Meier survival analysis demonstrated significantly higher cumulative mortality in higher RAR quartiles, with Q4 demonstrating the greatest cumulative 28-day hospital mortality (Figure 3). Similar trends were also observed for 90-day mortality (Figure 3).

**Table 2:** Odd ratio (OR) and β [95% confidence intervals (CI)] for mortality, and length of ICU and hospital stay across groups of RAR.

Variable	Model I		Model II		Model III	
	OR/*β* (95 % CI)	*p*-value	OR/*β* (95% CI)	*p*-value	OR/*β* (95% CI)	*p*-value
28-day hospital mortality						
RAR per 1-unit	1.26 (1.22, 1.30)	<0.0001	1.27 (1.23, 1.31)	<0.0001	1.20 (1.15, 1.24)	<0.0001
per SD	1.62 (1.53, 1.73)	<0.0001	1.65 (1.55, 1.76)	<0.0001	1.46 (1.35, 1.59)	<0.0001
Quartile 1	Ref		Ref		Ref	
Quartile 2	1.40 (1.11, 1.77)	0.0048	1.40 (1.10, 1.77)	0.0053	1.23 (0.95, 1.60)	0.1120
Quartile 3	2.36 (1.90, 2.94)	<0.0001	2.37 (1.90, 2.95)	<0.001	1.82 (1.41, 2.33)	<0.0001
Quartile 4	4.18 (3.39, 5.15)	<0.0001	4.33 (3.51, 5.34)	<0.0001	2.95 (2.28, 3.80)	<0.0001
*p* for trend		<0.0010		<0.0010		<0.0010
ICU mortality						
RAR per 1-unit	1.22 (1.17, 1.26)	<0.0001	1.22 (1.18, 1.26)	<0.0001	1.13 (1.08, 1.18)	<0.0001
per SD	1.51 (1.40, 1.62)	<0.0001	1.62 (1.41, 1.63)	<0.0001	1.29 (1.18, 1.42)	<0.0001
Quartile 1	Ref		Ref		Ref	
Quartile 2	1.29 (0.97, 1.70)	0.0786	1.28 (0.97, 1.69)	0.0849	1.05 (0.77, 1.43)	0.7741
Quartile 3	1.78(1.37, 2.33)	<0.0001	1.78(1.37, 2.33)	<0.001	1.28 (0.94, 1.73)	0.1129
Quartile 4	3.43 (2.68, 4.38)	<0.0001	3.46 (2.70, 4.43)	<0.0001	2.06 (1.52, 2.78)	<0.0001
*p* for trend		<0.0010		<0.0010		<0.0010
90-day hospital mortality						
RAR per 1-unit	1.27 (1.23, 1.30)	<0.0001	1.28 (1.24, 1.31)	<0.0001	1.21 (1.16, 1.25)	<0.0001
per SD	1.64 (1.54, 1.75)	<0.0001	1.67 (1.57, 1.78)	<0.0001	1.49 (1.37, 1.61)	<0.0001
Quartile 1	Ref		Ref		Ref	
Quartile 2	1.43 (1.14, 1.81)	0.0025	1.43 (1.13, 1.81)	0.0028	1.26 (0.98, 1.64)	0.0751
Quartile 3	2.38 (1.91, 2.96)	<0.0001	2.39 (1.92, 2.98)	<0.0001	1.83 (1.43, 2.36)	<0.0001
Quartile 4	4.29 (3.49, 5.29)	<0.0001	4.44 (3.60, 5.47)	<0.0001	3.03 (2.35, 3.90)	<0.0001
*p* for trend		<0.0010		<0.0010		<0.0010
Length of ICU stay						
RAR per 1-unit	0.24 (0.17, 0.31)	<0.0001	0.22 (0.15, 0.29)	<0.0001	0.17 (0.09, 0.25)	<0.0001
per SD	0.50 (0.35, 0.65)	<0.0001	0.47 (0.32, 0.62)	<0.0001	0.35 (0.18, 0.52)	<0.0001
Quartile 1	Ref		Ref		Ref	
Quartile 2	0.55 (0.13, 0.96)	0.0099	0.57 (0.16, 0.99)	0.0071	0.34 (−0.08, 0.77)	0.1149
Quartile 3	0.68 (0.26, 1.09)	0.0015	0.67 (0.26, 1.09)	0.0014	0.33 (−0.11, 0.77)	0.1446
Quartile 4	1.36 (0.94, 1.78)	<0.0001	1.30 (0.88, 1.71)	<0.0001	0.89 (0.42, 1.36)	0.0002
*p* for trend		<0.0010		<0.0010		<0.0010
Length of hospital stay						
RAR per 1-unit	0.38 (0.27, 0.48)	<0.0001	0.35 (0.24, 0.45)	<0.0001	0.30 (0.18, 0.42)	<0.0001
per SD	0.79 (0.57, 1.01)	<0.0001	0.73 (0.51, 0.95)	<0.0001	0.63 (0.38, 0.89)	<0.0001
Quartile 1	Ref		Ref		Ref	
Quartile 2	1.10 (0.48, 1.72)	0.0005	1.14 (0.52, 1.75)	0.0003	0.86 (0.22, 1.51)	0.0091
Quartile 3	1.50 (0.88, 2.12)	<0.0001	1.50 (0.88, 2.11)	<0.0001	1.04 (0.37, 1.71)	0.0023
Quartile 4	2.17 (1.55, 2.79)	<0.0001	2.03 (1.42, 2.65)	<0.0001	1.64 (0.93, 2.36)	<0.0001
*p* for trend		<0.0010		<0.0010		<0.0010

Model I is the crude model. Model II is adjusted for age, gender, ethnicity. Model III is adjusted for Model II plus BMI, SAPS II, SOFA score, ventilation, dialysis, vasopressor use, hepatic failure, metastatic cancer, immunosuppression, DM, COPD, CHF, AMI, infection site, BUN, creatinine, bicarbonate, glucose, ALT, AST, platelets, hemoglobin and WBC.

### 3.3 Subgroup analysis and sensitivity analysis

**Figure 4:**
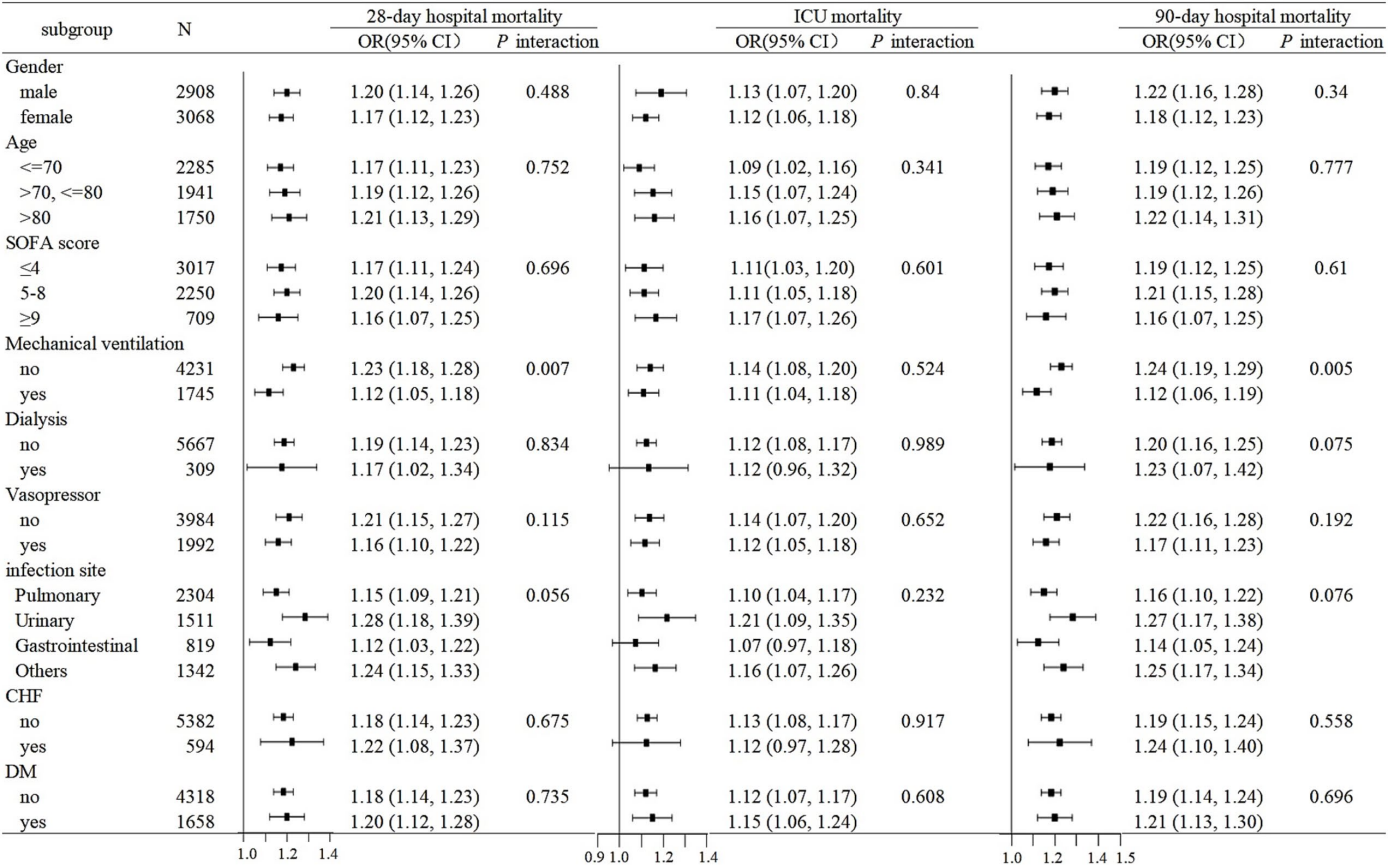
Subgroup analyses for the association of RAR with 28-day hospital mortality, ICU mortality, and 90-day hospital mortality.

As illustrated in Figure 4, subgroup analyses stratified by gender, age, SOFA score, mechanical ventilation, dialysis, vasopressor use, infection site, CHF, and DM demonstrated consistent associations between RAR and mortality risks across most strata (*P*-interaction >0.05; Figure 4). Notably, while a statistically significant interaction was observed for mechanical ventilation status (*P* interaction<0.05), the direction of effect remained concordant between ventilated and non-ventilated patients.

To ensure the robustness of our findings, several sensitivity analyses were performed. Initially, we excluded patients with missing data, and similar results were obtained, with the association between RAR and clinical outcomes in elderly sepsis patients remaining statistically significant (Supplementary Table S1). Additionally, after excluding patients who received red blood cell transfusion, plasma, or human serum albumin within 48 h prior to ICU admission, the sensitivity analysis yielded results analogous to the primary findings (Supplementary Table S2). Lastly, separate multivariable regression analyses were performed for patients with anemia and those without anemia, both demonstrating consistent associations between RAR and clinical outcomes in elderly sepsis patients (Supplementary Tables S3, S4).

## 4 Discussion

The present study aims to investigate the association between RAR and clinical outcomes in elderly sepsis patients. Although prior research has explored the association between RAR and prognostic outcomes in general adult patients, this is the first study to focus exclusively on elderly sepsis patients. Our primary finding demonstrates that an elevated baseline RAR, measured upon ICU admission, is independently associated with adverse clinical outcomes in this patient population. Therefore, RAR may serve as a valuable tool for early risk stratification and guiding personalized therapeutic interventions in elderly patients with sepsis.

Emerging evidence from recent investigations suggests that elevated RAR levels are significantly associated with increased mortality risk and poor prognosis across diverse disease conditions (21–23), highlighting its clinical utility in assessing disease outcomes. In the context of sepsis, several studies have further elucidated this relationship. For instance, analyses of data from the MIMIC database by Xu et al. and Ma et al. identified a positive association between elevated RAR and 28-day mortality, 90-day mortality, and in-hospital mortality in adult sepsis patients (27, 28). Jing et al. extended these observations to pediatric sepsis, demonstrating that higher RAR levels were associated with increased short- and long-term mortality rates (29). Notably, their study also found that RAR had superior predictive value for sepsis incidence and 28-day mortality compared to RDW or albumin alone. Longitudinal analyses by Tan et al. further revealed that RAR was positively associated with an increased risk of rehospitalization and rehospitalization all-cause mortality among adult sepsis survivors (30). The prognostic significance of RAR has been further validated in a recent large-scale critical care cohort encompassing sepsis and other ICU populations, demonstrating particularly heightened mortality risk at RAR values above 5.0 (31). Collectively, these findings emphasize the clinical relevance of RAR in both adult and pediatric sepsis populations. Nonetheless, the association between RAR and prognostic outcomes in elderly patients with sepsis remains underexplored.

In this study, we analyzed 5,976 sepsis patients aged 60 years and over admitted to the ICU. Multivariable regression analyses revealed that RAR, whether considered as a continuous variable or a categorical variable, was independently associated with an increased risk of 28-day hospital mortality, ICU mortality, and 90-day hospital mortality, as well as prolonged ICU and hospital length of stay. Generalized additive model revealed a linear dose–response relationship, demonstrating that increasing RAR levels were associated with higher mortality risk. Kaplan–Meier analysis further supported these findings, showing elevated mortality in patients with higher RAR levels compared to those with lower RAR levels. Subgroup analysis confirmed the consistency of these associations across various patient subgroups, reinforcing the robustness of our findings. Notably, we observed that elderly patients with elevated RAR had higher SOFA score, and a greater proportion required vasopressors and ventilator use, suggesting a potential link between RAR and disease severity in this patient population.

The precise mechanisms underlying the association between elevated RAR and increased mortality in elderly sepsis patients remain incompletely understood; however, several plausible explanations have been proposed. In sepsis, systemic inflammation and oxidative stress disrupt erythrocyte homeostasis, leading to impaired erythropoiesis, increased erythrocyte apoptosis, and the formation of heterogeneous erythrocyte populations with variable sizes and lifespans (32–34). Thus, this erythrocyte heterogeneity, reflected by elevated RDW, is a marker of systemic inflammation, oxidative stress, and impaired erythropoiesis, all of which are central to the pathophysiology of sepsis. Concurrently, systemic inflammation and oxidative stress during sepsis contribute to reduced serum albumin levels through multiple interconnected mechanisms, such as suppressed albumin synthesis, increased vascular permeability leading to albumin leakage, and accelerated protein catabolism due to a hypermetabolic state (17, 35, 36). As a composite index integrating RDW and albumin, RAR may comprehensively reflect the multifaceted nature of sepsis, including systemic inflammation, oxidative stress, impaired erythropoiesis, and malnutrition. Further research is required to fully elucidate the mechanistic pathways linking RAR to clinical outcomes.

In contrast to previous studies primarily focusing on adult sepsis patients, our research specifically targets elderly patients with sepsis. The incidence and mortality rates of sepsis in this demographic remain persistently high; however, reliable and easily accessible indicators for early risk stratification are still lacking. Our findings reveal an independent association between RAR and sepsis outcomes in older adults, highlighting RAR as an innovative marker that facilitates the early identification of high-risk patients and enables personalized therapeutic interventions. This addresses unmet clinical needs in the management of geriatric sepsis. Importantly, RAR employs routine laboratory parameters without requiring specialized assays, making it a cost-effective tool that aligns well with resource-conscious healthcare systems.

However, several limitations of this study should be acknowledged. First, the retrospective design, despite rigorous adjustments for potential confounders and comprehensive subgroup analyses using the eICU database, may still be prone to selection bias and unmeasured confounding factors. Second, although the analysis included baseline RAR values at ICU admission, it did not assess the dynamic changes in RAR over time, which could offer additional insights into sepsis outcomes. Third, relevant factors influencing RDW levels, such as therapeutic interventions like erythropoietin administration, iron supplementation, and vitamin B12 therapy, were not evaluated. Therefore, while our findings suggest a potential association, causal inference requires further validation.

## 5 Conclusion

This study demonstrates that elevated baseline RAR at ICU admission is independently associated with increased mortality risk and prolonged hospitalization in elderly sepsis patients. As a composite biomarker integrating routinely available laboratory parameters, RAR could serve as a pragmatic and cost-effective tool for early risk stratification, enabling timely identification of high-risk patients and guiding personalized therapeutic strategies to mitigate adverse outcomes in elderly sepsis patients.

## Data Availability

The original contributions presented in the study are included in the article/Supplementary material, further inquiries can be directed to the corresponding author/s.
