# Supplementary material for: Association between red cell distribution width to albumin ratio and clinical outcomes in elderly patients with sepsis: a cohort study
Source: Front Nutr. 2025 Sep 2;12:1617199. doi: 10.3389/fnut.2025.1617199 (PMC12436393; doi:10.3389/fnut.2025.1617199)
Supplement: Supplementary file 1 [file Table_1.docx]

| **variable** | **Model Ⅰ** | | **Model Ⅱ** | | **Model Ⅲ** | |
| --- | --- | --- | --- | --- | --- | --- |
|  | **OR**（95% CI） | ***p***-value | **OR**（95% CI） | ***p***-value | **OR**（95% CI） | ***p***-value |
| **28-day hospital mortality** |  |  |  |  |  |  |
| RAR per 1-unit | 1.26 (1.22, 1.30) | <0.0001 | 1.27 (1.23, 1.31) | <0.0001 | 1.19 (1.14, 1.24) | <0.0001 |
| per SD | 1.62 (1.53, 1.73) | <0.0001 | 1.66 (1.55, 1.77) | <0.0001 | 1.44 (1.32, 1.58) | <0.0001 |
| Quartile1 | Ref |  | Ref |  | Ref |  |
| Quartile2 | 1.40 (1.11, 1.77) | 0.0048 | 1.39 (1.09, 1.76) | 0.0069 | 1.26 (0.94, 1.69) | 0.1174 |
| Quartile3 | 2.36 (1.90, 2.94) | <0.0001 | 2.37 (1.90, 2.95) | <0.0001 | 1.82 (1.37, 2.41) | <0.0001 |
| Quartile4 | 4.18 (3.39, 5.15) | <0.0001 | 4.32 (3.50, 5.34) | <0.0001 | 2.95 (2.21, 3.92) | <0.0001 |
| *p* for trend |  | < 0.0010 |  | < 0.0010 |  | < 0.0010 |
| **ICU mortality** |  |  |  |  |  |  |
| RAR per 1-unit | 1.22 (1.17, 1.26) | <0.0001 | 1.22 (1.18, 1.27) | <0.0001 | 1.10 (1.05, 1.16) | 0.0002 |
| per SD | 1.51 (1.40, 1.62) | <0.0001 | 1.53 (1.42, 1.64) | <0.0001 | 1.23 (1.10, 1.37) | 0.0002 |
| Quartile1 | Ref |  | Ref |  | Ref |  |
| Quartile2 | 1.29 (0.97, 1.70) | 0.0786 | 1.26 (0.95, 1.67) | 0.1097 | 1.03 (0.73, 1.45) | 0.8725 |
| Quartile3 | 1.78 (1.37, 2.33) | <0.0001 | 1.77 (1.36, 2.32) | <0.0001 | 1.14 (0.82, 1.60) | 0.4385 |
| Quartile4 | 3.43 (2.68, 4.38) | <0.0001 | 3.49 (2.72, 4.47) | <0.0001 | 1.87 (1.35, 2.61) | 0.0002 |
| *p* for trend |  | < 0.001 |  | < 0.001 |  | < 0.001 |
| **90-day hospital mortality** |  |  |  |  |  |  |
| RAR per 1-unit | 1.27 (1.23, 1.30) | <0.0001 | 1.28 (1.24, 1.32) | <0.0001 | 1.20 (1.15, 1.26) | <0.0001 |
| per SD | 1.64 (1.54, 1.75) | <0.0001 | 1.67 (1.57, 1.78) | <0.0001 | 1.48 (1.35, 1.62) | <0.0001 |
| Quartile1 | Ref |  | Ref |  | Ref |  |
| Quartile2 | 1.44 (1.14, 1.81) | 0.0024 | 1.42 (1.12, 1.80) | 0.0033 | 1.30 (0.98, 1.74) | 0.0733 |
| Quartile3 | 2.37 (1.90, 2.95) | <0.0001 | 2.38 (1.91, 2.96) | <0.0001 | 1.84 (1.39, 2.44) | <0.0001 |
| Quartile4 | 4.31 (3.50, 5.31) | <0.0001 | 4.45 (3.60, 5.49) | <0.0001 | 3.06 (2.30, 4.06) | <0.0001 |
| *p* for trend |  | < 0.0010 |  | < 0.0010 |  | < 0.0010 |
| **Length of ICU stay** |  |  |  |  |  |  |
| RAR per 1-unit | 0.24 (0.17, 0.31) | <0.0001 | 0.23 (0.16, 0.30) | <0.0001 | 0.20 (0.10, 0.30) | <0.0001 |
| per SD | 0.50 (0.35, 0.65) | <0.0001 | 0.49 (0.34, 0.63) | <0.0001 | 0.42 (0.21, 0.62) | <0.0001 |
| Quartile1 | Ref |  | Ref |  | Ref |  |
| Quartile2 | 0.55 (0.13, 0.96) | 0.0099 | 0.60 (0.18, 1.01) | 0.0051 | 0.39 (-0.13, 0.92) | 0.1384 |
| Quartile3 | 0.68 (0.26, 1.09) | 0.0015 | 0.71 (0.29, 1.12) | 0.0009 | 0.39 (-0.14, 0.93) | 0.1483 |
| Quartile4 | 1.36 (0.94, 1.78) | <0.0001 | 1.33 (0.91, 1.75) | <0.0001 | 1.04 (0.47, 1.61) | 0.0004 |
| *p* for trend |  | < 0.0010 |  | < 0.0010 |  | < 0.0010 |
| **Length of hospital stay** |  |  |  |  |  |  |
| RAR per 1-unit | 0.38 (0.27, 0.48) | <0.0001 | 0.35 (0.25, 0.46) | <0.0001 | 0.34 (0.20, 0.48) | <0.0001 |
| per SD | 0.79 (0.57, 1.01) | <0.0001 | 0.74 (0.52, 0.96) | <0.0001 | 0.72 (0.42, 1.01) | <0.0001 |
| Quartile1 | Ref |  | Ref |  | Ref |  |
| Quartile2 | 1.10 (0.48, 1.72) | 0.0005 | 1.16 (0.54, 1.78) | 0.0002 | 0.73 (-0.02, 1.48) | 0.0571 |
| Quartile3 | 1.50 (0.88, 2.12) | <0.0001 | 1.51 (0.89, 2.13) | <0.0001 | 0.96 (0.19, 1.73) | 0.0141 |
| Quartile4 | 2.17 (1.55, 2.79) | <0.0001 | 2.06 (1.43, 2.68) | <0.0001 | 1.72 (0.89, 2.54) | <0.0001 |
| *p* for trend |  | < 0.0010 |  | < 0.0010 |  | < 0.0010 |

**Table S1** Odd ratio (OR) and β [95% confidence intervals (CI)] for mortality, and length of ICU/hospital stay across groups of RAR (with missing data).

**Table S2** Odd ratio (OR) and β [95% confidence intervals (CI)] for mortality, and length of ICU/hospital stay across groups of RAR (excluding patients who received blood transfusions or albumin infusion).

| **variable** | **Model Ⅰ** | | **Model Ⅱ** | | **Model Ⅲ** | |
| --- | --- | --- | --- | --- | --- | --- |
|  | **OR (95% CI)** | ***p*-value** | **OR (95% CI)** | ***p*-value** | **OR (95% CI)** | ***p*-value** |
| **28-day hospital mortality** |  |  |  |  |  |  |
| RAR per 1-unit | 1.26 (1.22, 1.30) | <0.0001 | 1.27 (1.23, 1.31) | <0.0001 | 1.22 (1.17, 1.26) | <0.0001 |
| per SD | 1.62 (1.53, 1.73) | <0.0001 | 1.65 (1.55, 1.76) | <0.0001 | 1.51 (1.40, 1.63) | <0.0001 |
| Quartile 1 | Ref |  | Ref |  | Ref |  |
| Quartile 2 | 1.40 (1.11, 1.78) | 0.0046 | 1.40 (1.11, 1.77) | 0.0051 | 1.28 (0.99, 1.64) | 0.0556 |
| Quartile 3 | 2.36 (1.90, 2.94) | <0.0001 | 2.37 (1.91, 2.96) | <0.0001 | 2.03 (1.59, 2.58) | <0.0001 |
| Quartile 4 | 4.18 (3.39, 5.15) | <0.0001 | 4.33 (3.51, 5.34) | <0.0001 | 3.28 (2.57, 4.19) | <0.0001 |
| *p* for trend |  | <0.0001 |  | <0.0001 |  | <0.0001 |
| **ICU mortality** |  |  |  |  |  |  |
| RAR per 1-unit | 1.22 (1.17, 1.26) | <0.0001 | 1.22 (1.18, 1.26) | <0.0001 | 1.14 (1.09, 1.19) | <0.0001 |
| per SD | 1.51 (1.40, 1.62) | <0.0001 | 1.52 (1.41, 1.63) | <0.0001 | 1.31 (1.20, 1.43) | <0.0001 |
| Quartile 1 | Ref |  | Ref |  | Ref |  |
| Quartile 2 | 1.29 (0.97, 1.71) | 0.0769 | 1.28 (0.97, 1.70) | 0.0832 | 1.10 (0.81, 1.48) | 0.5511 |
| Quartile 3 | 1.79 (1.37, 2.33) | <0.0001 | 1.78 (1.37, 2.33) | <0.0001 | 1.33 (0.99, 1.78) | 0.0566 |
| Quartile 4 | 3.43 (2.68, 4.38) | <0.0001 | 3.46 (2.70, 4.43) | <0.0001 | 2.18 (1.63, 2.91) | <0.0001 |
| *p* for trend |  | <0.0001 |  | <0.0001 |  | <0.0001 |
| **90-day hospital mortality** |  |  |  |  |  |  |
| RAR per 1-unit | 1.27 (1.23, 1.30) | <0.0001 | 1.28 (1.24, 1.32) | <0.0001 | 1.23 (1.18, 1.27) | <0.0001 |
| per SD | 1.64 (1.54, 1.75) | <0.0001 | 1.67 (1.57, 1.78) | <0.0001 | 1.53 (1.42, 1.65) | <0.0001 |
| Quartile 1 | Ref |  | Ref |  | Ref |  |
| Quartile 2 | 1.43 (1.13, 1.80) | 0.0028 | 1.42 (1.13, 1.80) | 0.0032 | 1.31 (1.02, 1.68) | 0.0354 |
| Quartile 3 | 2.38 (1.92, 2.96) | <0.0001 | 2.39 (1.92, 2.98) | <0.0001 | 2.06 (1.62, 2.62) | <0.0001 |
| Quartile 4 | 4.29 (3.49, 5.29) | <0.0001 | 4.44 (3.60, 5.47) | <0.0001 | 3.40 (2.67, 4.33) | <0.0001 |
| *p* for trend |  | <0.0001 |  | <0.0001 |  | <0.0001 |
| **Length of ICU stay** |  |  |  |  |  |  |
| RAR per 1-unit | 0.24 (0.17, 0.31) | <0.0001 | 0.22 (0.15, 0.29) | <0.0001 | 0.15 (0.07, 0.23) | 0.0001 |
| per SD | 0.50 (0.35, 0.65) | <0.0001 | 0.47 (0.32, 0.62) | <0.0001 | 0.32 (0.16, 0.48) | 0.0001 |
| Quartile 1 | Ref |  | Ref |  | Ref |  |
| Quartile 2 | 0.55 (0.13, 0.96) | 0.0101 | 0.57 (0.15, 0.98) | 0.0072 | 0.45 (0.04, 0.87) | 0.03 |
| Quartile 3 | 0.68 (0.26, 1.09) | 0.0014 | 0.68 (0.26, 1.09) | 0.0014 | 0.43 (0.01, 0.86) | 0.0452 |
| Quartile 4 | 1.36 (0.94, 1.78) | <0.0001 | 1.30 (0.88, 1.71) | <0.0001 | 0.84 (0.39, 1.29) | 0.0003 |
| *p* for trend |  | <0.0001 |  | <0.0001 |  | <0.0001 |
| **Length of hospital stay** |  |  |  |  |  |  |
| RAR per 1-unit | 0.38 (0.27, 0.48) | <0.0001 | 0.35 (0.24, 0.45) | <0.0001 | 0.30 (0.19, 0.42) | <0.0001 |
| per SD | 0.79 (0.57, 1.01) | <0.0001 | 0.73 (0.51, 0.95) | <0.0001 | 0.64 (0.39, 0.88) | <0.0001 |
| Quartile 1 | Ref |  | Ref |  | Ref |  |
| Quartile 2 | 1.09 (0.47, 1.71) | 0.0006 | 1.13 (0.51, 1.74) | 0.0003 | 1.06 (0.44, 1.68) | 0.0009 |
| Quartile 3 | 1.51 (0.89, 2.13) | <0.0001 | 1.50 (0.89, 2.12) | <0.0001 | 1.35 (0.70, 1.99) | <0.0001 |
| Quartile 4 | 2.17 (1.55, 2.79) | <0.0001 | 2.03 (1.42, 2.65) | <0.0001 | 1.72 (1.03, 2.40) | <0.0001 |
| *p* for trend |  | <0.0001 |  | <0.0001 |  | <0.0001 |

**Table S3**  Odd ratio (OR) and β [95% confidence intervals (CI)] for mortality, and length of ICU/hospital stay across groups of RAR (patients with anemia).

| **variable** | | **Model Ⅰ** | | | | **Model Ⅱ** | | | | **Model Ⅲ** | | | |
| --- | --- | --- | --- | --- | --- | --- | --- | --- | --- | --- | --- | --- | --- |
|  |  | **OR**（**95% CI**） | | ***p*-value** | | **OR**（**95% CI**） | | ***p*-value** | | **OR**（**95% CI**） | | ***p*-value** | |
| **28-day hospital mortality** | |  | |  | |  | |  | |  | |  | |
| RAR per 1-unit | | 1.25 (1.21, 1.29) | | <0.0001 | | 1.26 (1.22, 1.30) | | <0.0001 | | 1.20 (1.15, 1.25) | | <0.0001 | |
| per SD | | 1.59 (1.49, 1.71) | | <0.0001 | | 1.62 (1.52, 1.74) | | <0.0001 | | 1.47 (1.36, 1.59) | | <0.0001 | |
| Quartile1 | | Ref | |  | | Ref | |  | | Ref | |  | |
| Quartile2 | | 1.36 (1.05, 1.75) | | 0.0184 | | 1.36 (1.05, 1.75) | | 0.0186 | | 1.17 (0.89, 1.53) | | 0.2538 | |
| Quartile3 | | 2.33 (1.84, 2.95) | | <0.0001 | | 2.36 (1.86, 2.99) | | <0.0001 | | 1.93 (1.49, 2.49) | | <0.0001 | |
| Quartile4 | | 4.02 (3.20, 5.03) | | <0.0001 | | 4.16 (3.31, 5.22) | | <0.0001 | | 2.97 (2.30, 3.85) | | <0.0001 | |
| *p* for trend | |  | | < 0.0010 | |  | | < 0.0010 | |  | | < 0.0010 | |
| **ICU mortality** | |  | |  | |  | |  | |  | |  | |
| RAR per 1-unit | | 1.20 (1.15, 1.24) | | <0.0001 | | 1.20 (1.16, 1.25) | | <0.0001 | | 1.12 (1.07, 1.17) | | <0.0001 | |
| per SD | | 1.46 (1.35, 1.58) | | <0.0001 | | 1.47 (1.36, 1.60) | | <0.0001 | | 1.26 (1.14, 1.39) | | <0.0001 | |
| Quartile1 | | Ref | |  | | Ref | |  | | Ref | |  | |
| Quartile2 | | 1.23 (0.91, 1.66) | | 0.181 | | 1.23 (0.91, 1.66) | | 0.1846 | | 1.00 (0.72, 1.38) | | 0.9883 | |
| Quartile3 | | 1.77 (1.33, 2.35) | | <0.0001 | | 1.77 (1.33, 2.36) | | <0.0001 | | 1.29 (0.95, 1.76) | | 0.1074 | |
| Quartile4 | | 3.19 (2.45, 4.16) | | <0.0001 | | 3.22 (2.47, 4.20) | | <0.0001 | | 1.94 (1.43, 2.63) | | <0.0001 | |
| *p* for trend | |  | | < 0.0010 | |  | | < 0.0010 | |  | | < 0.0010 | |
| **90-day hospital mortality** | |  | |  | |  | |  | |  | |  | |
| RAR per 1-unit | | 1.25 (1.21, 1.29) | | <0.0001 | | 1.26 (1.22, 1.31) | | <0.0001 | | 1.21 (1.16, 1.26) | | <0.0001 | |
| per SD | | 1.61 (1.51, 1.73) | | <0.0001 | | 1.64 (1.53, 1.76) | | <0.0001 | | 1.50 (1.38, 1.62) | | <0.0001 | |
| Quartile1 | | Ref | |  | | Ref | |  | | Ref | |  | |
| Quartile2 | | 1.40 (1.08, 1.80) | | 0.0096 | | 1.40 (1.08, 1.80) | | 0.0097 | | 1.22 (0.93, 1.59) | | 0.1499 | |
| Quartile3 | | 2.35 (1.86, 2.98) | | <0.0001 | | 2.38 (1.88, 3.02) | | <0.0001 | | 1.97 (1.53, 2.54) | | <0.0001 | |
| Quartile4 | | 4.12 (3.29, 5.16) | | <0.0001 | | 4.27 (3.40, 5.35) | | <0.0001 | | 3.09 (2.39, 3.99) | | <0.0001 | |
| *p* for trend | |  | | < 0.0010 | |  | | < 0.0010 | |  | | < 0.0010 | |
| **Length of ICU stay** | |  | |  | |  | |  | |  | |  | |
| RAR per 1-unit | | 0.24 (0.16, 0.32) | | <0.0001 | | 0.22 (0.15, 0.30) | | <0.0001 | | 0.14 (0.05, 0.22) | | 0.0012 | |
| per SD | | 0.50 (0.34, 0.67) | | <0.0001 | | 0.47 (0.31, 0.64) | | <0.0001 | | 0.29 (0.11, 0.46) | | 0.0012 | |
| Quartile1 | | Ref | |  | | Ref | |  | | Ref | |  | |
| Quartile2 | | 0.47 (0.01, 0.93) | | 0.0464 | | 0.47 (0.01, 0.93) | | 0.0445 | | 0.37 (-0.09, 0.82 | | ) 0.1120 | |
| Quartile3 | | 0.76 (0.30, 1.22) | | 0.0013 | | 0.73 (0.28, 1.19) | | 0.0017 | | 0.45 (-0.01, 0.91 | | ) 0.0576 | |
| Quartile4 | | 1.40 (0.94, 1.86) | | <0.0001 | | 1.33 (0.87, 1.79) | | <0.0001 | | 0.82 (0.33, 1.31) | | 0.0011 | |
| *p* for trend | |  | | < 0.0010 | |  | | < 0.0010 | |  | | < 0.0010 | |
| **Length of hospital stay** | |  | |  | |  | |  | |  | |  | |
| RAR per 1-unit | | 0.35 (0.24, 0.47) | | <0.0001 | | 0.32 (0.21, 0.43) | | <0.0001 | | 0.28 (0.16, 0.40) | | <0.0001 | |
| per SD | | 0.75 (0.51, 0.99) | | <0.0001 | | 0.68 (0.44, 0.92) | | <0.0001 | | 0.59 (0.33, 0.85) | | <0.0001 | |
| Quartile1 | | Ref | |  | | Ref | |  | | Ref | |  | |
| Quartile2 | | 0.75 (0.08, 1.42) | | 0.0287 | | 0.76 (0.09, 1.43) | | 0.0269 | | 0.70 (0.03, 1.37) | | 0.0393 | |
| Quartile3 | | 1.57 (0.90, 2.24) | | <0.0001 | | 1.53 (0.86, 2.20) | | <0.0001 | | 1.32 (0.64, 2.01) | | 0.0002 | |
| Quartile4 | | 1.99 (1.32, 2.67) | | <0.0001 | | 1.85 (1.18, 2.52) | | <0.0001 | | 1.55 (0.83, 2.28) | | <0.0001 | |
| *p* for trend | |  | | < 0.0010 | |  | | < 0.0010 | |  | | < 0.0010 | |
| **variable** | | **Model Ⅰ** | | | | **Model Ⅱ** | | | | **Model Ⅲ** | | | |
|  |  | **OR**（**95% CI**） | | ***p*-value** | | **OR**（**95% CI**） | | ***p*-value** | | **OR**（**95% CI**） | | ***p*-value** | |
| **28-day hospital mortality** | |  | |  | |  | |  | |  | |  | |
| RAR per 1-unit | | 1.46 (1.33, 1.61) | | <0.0001 | | 1.48 (1.34, 1.63) | | <0.0001 | | 1.32 (1.17, 1.50) | | <0.0001 | |
| per SD | | 1.88 (1.60, 2.20) | | <0.0001 | | 1.91 (1.62, 2.24) | | <0.0001 | | 1.59 (1.29, 1.96) | | <0.0001 | |
| Quartile1 | | Ref | |  | | Ref | |  | | Ref | |  | |
| Quartile2 | | 1.78 (0.93, 3.39) | | 0.0801 | | 1.74 (0.91, 3.32) | | 0.093 | | 1.49 (0.72, 3.09) | | 0.2788 | |
| Quartile3 | | 2.99 (1.63, 5.48) | | 0.0004 | | 2.91 (1.58, 5.35) | | 0.0006 | | 2.63 (1.32, 5.23) | | 0.0058 | |
| Quartile4 | | 6.03 (3.40, 10.71) | | <0.0001 | | 6.19 (3.48, 11.01) | | <0.0001 | | 4.32 (2.18, 8.56) | | <0.0001 | |
| *p* for trend | |  | | < 0.0010 | |  | | < 0.0010 | |  | | < 0.0010 | |
| **ICU mortality** | |  | |  | |  | |  | |  | |  | |
| RAR per 1-unit | | 1.42 (1.28, 1.58) | | <0.0001 | | 1.43 (1.28, 1.59) | | <0.0001 | | 1.19 (1.04, 1.37) | | 0.0108 | |
| per SD | | 1.79 (1.50, 2.13) | | <0.0001 | | 1.80 (1.51, 2.15) | | <0.0001 | | 1.34 (1.07, 1.68) | | 0.0108 | |
| Quartile1 | | Ref | |  | | Ref | |  | | Ref | |  | |
| Quartile2 | | 1.49 (0.68, 3.28) | | 0.3199 | | 1.49 (0.68, 3.28) | | 0.3227 | | 1.15 (0.46, 2.88) | | 0.7659 | |
| Quartile3 | | 2.62 (1.26, 5.42) | | 0.0097 | | 2.59 (1.25, 5.37) | | 0.0107 | | 2.19 (0.95, 5.04) | | 0.0668 | |
| Quartile4 | | 4.49 (2.26, 8.92) | | <0.0001 | | 4.61 (2.32, 9.17) | | <0.0001 | | 2.08 (0.91, 4.73) | | 0.0815 | |
| *p* for trend | |  | | < 0.0010 | |  | | < 0.0010 | |  | | < 0.0010 | |
| **90-day hospital mortality** | |  | |  | |  | |  | |  | |  | |
| RAR per 1-unit | | 1.47 (1.34, 1.62) | | <0.0001 | | 1.48 (1.34, 1.64) | | <0.0001 | | 1.33 (1.17, 1.51) | | <0.0001 | |
| per SD | | 1.89 (1.61, 2.22) | | <0.0001 | | 1.92 (1.63, 2.25) | | <0.0001 | | 1.61 (1.30, 1.99) | | <0.0001 | |
| Quartile1 | | Ref | |  | | Ref | |  | | Ref | |  | |
| Quartile2 | | 1.78 (0.93, 3.39) | | 0.0801 | | 1.74 (0.91, 3.33) | | 0.092 | | 1.46 (0.71, 3.03) | | 0.3061 | |
| Quartile3 | | 3.07 (1.68, 5.63) | | 0.0003 | | 3.00 (1.63, 5.51) | | 0.0004 | | 2.68 (1.35, 5.31) | | 0.0048 | |
| Quartile4 | | 6.15 (3.47, 10.91) | | <0.0001 | | 6.29 (3.54, 11.17) | | <0.0001 | | 4.34 (2.20, 8.59) | | <0.0001 | |
| *p* for trend | |  | | < 0.0010 | |  | | < 0.0010 | |  | | < 0.0010 | |
| **Length of ICU stay** | |  | |  | |  | |  | |  | |  | |
| RAR per 1-unit | | 0.46 (0.25, 0.66) | | <0.0001 | | 0.45 (0.24, 0.65) | | <0.0001 | | 0.38 (0.15, 0.61) | | 0.0011 | |
| per SD | | 0.76 (0.42, 1.10) | | <0.0001 | | 0.74 (0.40, 1.08) | | <0.0001 | | 0.63 (0.25, 1.01) | | 0.0011 | |
| Quartile1 | | Ref | |  | | Ref | |  | | Ref | |  | |
| Quartile2 | | 0.73 (-0.24, 1.69) | | 0.1399 | | 0.78 (-0.18, 1.74) | | 0.1132 | | 0.43 (-0.53, 1.39) | | 0.3831 | |
| Quartile3 | | 1.20 (0.23, 2.17) | | 0.0152 | | 1.26 (0.30, 2.23) | | 0.0107 | | 0.84 (-0.14, 1.82) | | 0.0921 | |
| Quartile4 | | 2.04 (1.09, 3.00) | | <0.0001 | | 2.01 (1.05, 2.96) | | <0.0001 | | 1.38 (0.35, 2.42) | | 0.0087 | |
| *p* for trend | |  | | < 0.0010 | |  | | < 0.0010 | |  | | < 0.0010 | |
| **Length of hospital stay** | |  | |  | |  | |  | |  | |  | |
| RAR per 1-unit | | 0.74 (0.40, 1.08) | | <0.0001 | | 0.72 (0.38, 1.06) | | <0.0001 | | 0.61 (0.23, 1.00) | | 0.002 | |
| per SD | | 1.22 (0.66, 1.78) | | <0.0001 | | 1.19 (0.64, 1.75) | | <0.0001 | | 1.01 (0.37, 1.65) | | 0.002 | |
| Quartile1 | | Ref | |  | | Ref | |  | | Ref | |  | |
| Quartile2 | | 1.48 (-0.10, 3.06) | | 0.067 | | 1.58 (0.01, 3.16) | | 0.0493 | | 1.11 (-0.50, 2.73) | | 0.1779 | |
| Quartile3 | | 2.11 (0.51, 3.70) | | 0.0098 | | 2.25 (0.66, 3.84) | | 0.0057 | | 1.68 (0.03, 3.33) | | 0.0462 | |
| Quartile4 | | 3.40 (1.83, 4.97) | | <0.0001 | | 3.32 (1.76, 4.89) | | <0.0001 | | 2.50 (0.76, 4.23) | | 0.0049 | |
| *p* for trend | |  | | < 0.0010 | |  | | < 0.0010 | |  | | < 0.0010 | |

**Table S4**  Odd ratio (OR) and β [95% confidence intervals (CI)] for mortality, and length of ICU/hospital stay across groups of RAR (patients without anemia).
